# Coping Strategies in Stress Management and Burnout Prevention among Nurses

**DOI:** 10.1192/j.eurpsy.2026.11689

**Published:** 2026-07-31

**Authors:** J. Ben Khelifa, A. Fki, N. Gannoun, R. Nakhli, C. Sridi, F. Chelly, N. Belhadj, M. Maoua

**Affiliations:** 1 Clinical Psychology Unit, Occupational Medicine Department,; 2 Occupational Medicine Department, Sahloul University Hospital; 3 Faculty of Medicine of Sousse, University of Sousse; 4 Research Laboratory (LR19SP03), Sousse, Sousse, Tunisia

## Abstract

**Introduction:**

Occupational stress is increasingly recognized as a critical challenge in healthcare settings, with nurses being among the most exposed professionals. The demanding nature of their work, combined with high emotional involvement, can lead to elevated stress levels and eventually to burnout

**Objectives:**

This study aimed to explore coping strategies used by nurses in managing stress and preventing burnout.

**Methods:**

We conducted a cross-sectional descriptive and analytical study using a self-administered questionnaire and the WCC (Ways of Coping Checklist) scale. The survey involved 100 nurses working in various departments of Sahloul University Hospital during November and December 2024. Data analysis was performed using SPSS version 25.

**Results:**

The study population was predominantly female (69%), with a sex ratio of 0.44. Nearly three-quarters of participants (72%) were aged between 30 and 39 years, with a mean age of 35.18 years. About half of the respondents (49%) considered their work to be tiring, and 47% described it as exhausting. A high workload was reported as the main stress factor by 57% of participants, followed by emotional overload (29%). Only 10% of nurses had received specific training in stress management in addition to their basic education. Burnout was present in 88% of nurses According to the WCC scale, problem-focused coping was the preferred strategy, used by 61% of respondents.

**Conclusions:**

Our findings highlight the high prevalence of burnout symptoms among nurses and the limited availability of targeted training in stress management. Strengthening coping resources through structured interventions, continuous professional development, and organizational support is crucial to improve nurses’ well-being and prevent burnout in healthcare settings.

**Disclosure of Interest:**

None Declared

